# Compositional Analysis of Complex Mixtures using Automatic MicroED Data Collection

**DOI:** 10.1002/advs.202400081

**Published:** 2024-04-22

**Authors:** Johan Unge, Jieye Lin, Sara J Weaver, Ampon Sae Her, Tamir Gonen

**Affiliations:** ^1^ Department of Biological Chemistry University of California Los Angeles, 615 Charles E. Young Drive South Los Angeles California 90095 USA; ^2^ Department of Physiology University of California Los Angeles, 615 Charles E. Young Drive South Los Angeles California 90095 USA; ^3^ Howard Hughes Medical Institute University of California Los Angeles Los Angeles California 90095 USA

**Keywords:** analytical, automation, composition, CryoEM, high‐throughput, microcrystal electron diffraction, MicroED

## Abstract

Quantitative analysis of complex mixtures, including compounds having similar chemical properties, is demonstrated using an automatic and high throughput approach to microcrystal electron diffraction (MicroED). Compositional analysis of organic and inorganic compounds can be accurately executed without the need of diffraction standards. Additionally, with sufficient statistics, small amounts of compounds in mixtures can be reliably detected. These compounds can be distinguished by their crystal structure properties prior to structure solution. In addition, if the crystals are of good quality, the crystal structures can be generated on the fly, providing a complete analysis of the sample. MicroED is an effective method for analyzing the structural properties of sub‐micron crystals, which are frequently found in small‐molecule powders. By developing and using an automatic and high throughput approach to MicroED, and with the use of SerialEM for data collection, data from thousands of crystals allow sufficient statistics to detect even small amounts of compounds reliably.

## Graphical abstract

Quantitative analysis of complex mixtures, including chemically similar compounds, is demonstrated using an automatic and high throughput approach to MicroED. Compositional analysis can be accurately executed without the need of diffraction standards. Compounds are distinguished by their structure properties prior to structure solution. With sufficient statistics, small amounts of compounds in mixtures can be reliably detected. The crystal structures can be directly solved providing good quality data.

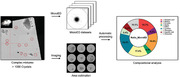

## 1 Introduction

Microcrystal electron diffraction (MicroED) is a cryoEM method for determining the 3D structure of inorganic, organic, or biological macromolecules.^[^ ^1^, ^2^ ^]^ Compared to X‐rays or neutrons, electrons exhibit a stronger interaction with the sample and cause considerably less damage per useful elastic scattering event. Thus, the optimal crystal size for MicroED is well below 1 µm^3^, and even crystals consisting of only a few layers can be used for structure determination.^[^ ^3^ ^]^ In fact, electron diffraction is currently the only method that can routinely produce a complete diffraction dataset for samples of this size and can be acquired with only picograms of a sample. This contrasts with X‐ray free‐electron laser (XFEL) serial crystallography and serial synchrotron crystallography (SSX), which require hundreds of thousands of crystals, with the crystals needing to be larger than 1 µm and 5 µm, respectively.^[^ ^4^, ^5^ ^]^ Ultimately, crystallographic techniques that use electrons, X‐rays, or neutrons are complementary because the beams interacting with the sample have different sensitivities for various elements. Moreover, due to their relatively larger cross‐section as compared with X‐rays, MicroED and neutron crystallography can often visualize hydrogen atoms.^[^ ^6^ ^]^ Finally, electron diffraction can provide information on charge.

Determining the composition of mixtures of compounds is a common and essential task that can be challenging as the components may have similar physical and chemical properties. There are several methods for determining the composition of mixtures, including chromatography, spectroscopy, and mass spectrometry.^[^ ^7^, ^8^, ^9^ ^]^ The gold standard for the analysis of crystalline powder has been powder X‐ray diffraction (PXRD), which is capable of identifying phases of mixtures with a detection limit of ≈5–10% for typical laboratory X‐ray diffractometers.^[^ ^10^ ^]^ The method is however limited to identification of compounds with a known crystal structure and diffraction; in fact, the diffraction of all components in the mixture needs to be simulated and compared with the experimental result in order to get unambiguous results. For complex materials peak overlay may present problems for phase analysis, in particular at high diffraction angles, due to the 1D d‐spacings analysis. In order to determine the structure of unknown compounds, a homogeneous sample is usually needed.

In recent years, MicroED has emerged as a promising technique for identifying and quantifying the components of complex mixtures, offering high resolution and sensitivity. Crystallization is itself a stereochemically discriminating process and naturally distinguishes between different isomers, including constitutional, conformational, geometric, diastereomer, and enantiomer isomers, based on their crystal structure properties rather than chemical properties.^[^ ^11^ ^]^ The structure of unknown well diffracting compounds is easily obtained with the lower limit of a few picograms of a sample consisting of a few nano sized crystals only, independent of further constituents of the sample. Even without solving the full crystal structure, the unit cell parameters and space group can be used as a signature of the crystal structure and its content. This means that most compounds can be distinguished by their unit cells, making compositional analysis of crystalline samples possible without collecting a full set of crystallographic data. Since determining the unit cell parameters is one of the first steps in structure determination and does not depend on solving the phase problem or collecting high‐resolution data, this approach can be used even when the diffraction qualities of the compounds are limited.

High throughput automation is increasingly essential in structural biology methods, particularly in MicroED, where multiple data sets are often required. Automatic data collection reduces manual labor, increases instrument usage, and allows larger data sets to be collected in the same time frame. This is especially important when many data sets must be merged to achieve higher completeness or when crystals are oriented preferentially on the grid. Additionally, studies have shown that automated approaches are effective in analyzing multiple phase systems and distinguishing different crystal forms. For instance, Wang et al. demonstrated the automated analysis of two zeolites using rotation electron diffraction.^[^ ^12^ ^]^ Smeets et al. used Instamatic to determine two structures from four phases in a multiple phase system.^[^ ^13^ ^]^ Jones et al. distinguished four natural products from a heterogeneous powder mixture,^[^ ^14^ ^]^ while Ge et al. identified two different zeolitic‐imidazolate frameworks from phase mixtures.^[^ ^15^ ^]^ Broadhurst et al. identified four forms of carbamazepine based on their unit‐cell dimensions and processed them to structures of the respective forms.^[^ ^16^ ^]^ Luo et al. automatically examined hundreds of crystals and identified five similar zeolite phases,^[^ ^17^ ^]^ and Sasaki et al. found two synthesized phases using SerialEM in diffraction mode,^[^ ^18^ ^]^ one of which led to a MicroED structure. These studies demonstrate the potential of high throughput automation in collecting and analyzing data in MicroED and other structural biology methods and highlight the ability of automated methods to accelerate the discovery of new crystal forms and improve our understanding of complex material structures.

In this work we first present a method for compositional analysis of complex mixtures comprising nine salts, saccharides, or amino acids per sample at varying percentages. The compounds in each group possess comparable physical and chemical characteristics such as weight, hydrophilicity, and charge distribution, which are standard distinguishing factors in most analytical methods. Some compounds have the same chemical formula but differ in stereochemistry (four compounds) or constitutional isomers (three compounds), making separation challenging for typical analysis methods. We developed a high throughput automatic MicroED approach to enable identification of all constituents and their relative ratios, mostly with high accuracy of just a few percent of their total composition. To demonstrate the extension of MicroED's capabilities using this approach, we applied the method to a salt mixture and identified all the ingredients in a mixture of nine compounds, with the smallest amount of constituents being around 3% by total mass. We also applied the method to drug formulations of aspirin and acetaminophen tablets. Among other crystalline ingredients, we identified the active ingredients with less than 2% errors in their mass ratios.

## 2 Results & Discussion

### 2.1 Identifying Compounds in a Complex Mixture using High Throughput Automatic MicroED

**Figure 1:**
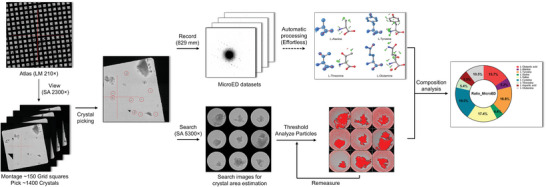
Workflow. The workflow for automatic MicroED data collection and processing involves several steps. First, a low‐magnification atlas is used to screen the grid containing a mixture of compounds, and suitable grid squares are selected for medium‐magnification montages. Crystals are then chosen and added to the list for data collection. Next, continuous rotation MicroED movie and an image of the crystal are collected automatically using SerialEM.^[^ ^19^, ^20^, ^21^ ^]^ The data is then processed automatically in real time. Crystal volume is estimated for compositional analysis. This workflow allows for efficient and automatic MicroED data collection and processing of thousands of data sets per night, reducing the need for human intervention and saving researchers' time while producing high‐quality data.

We developed new procedures in SerialEM and coupled them to a Python script that we developed to automatically collect and process MicroED data generated from them in real time. The method involves collecting an atlas montage of the entire grid, followed by medium‐magnification montages of the most promising grid squares and finally applying the data collection script to the crystals selected from the medium‐magnification montages (**Figure** **1**). We used this approach to determine the components in complex mixtures. We used several mixtures in this study as proof of principle. Mixture A contained several inorganic salts while mixtures B and C contained several saccharides and amino acids, respectively. These mixtures were analyzed using the pipeline to identify all their components. In addition to the manually prepared heterogeneous mixtures, we analyzed the components from the aspirin and acetaminophen mixtures which were ground from the commercial drugs. The crystalline ingredients were successfully identified and observed with promising consistency to the values listed in the drug information.

Mixture A was prepared with varying amounts of salts ranging from 3.7 to 29.3 mg, totaling to 112.0 mg (Table S3, Supporting Information) The mixture composed of Sodium bicarbonate (14.4% *v/v*), Sodium sulfate (21.3% *v/v*), Potassium sulfate (14.2% *v/v*), Magnesium sulfate heptahydrate (6.7% *v/v*), Sodium chloride (5.4% *v/v*), Calcium gluconate (5.9% *v/v*), Sodium citrate dihydrate (15.4% *v/v*), Calcium acetate monohydrate (4.7% *v/v*), Magnesium acetate tetrahydrate (12.0% *v/v*). When this mixture was loaded to the microscope, several crystals appeared “melted” as their edges were not sharp suggesting that somehow in the process hydration may have occurred which may lead to a loss in crystal order and resolution. Hydration could have happened in the test tube prior to grid preparation. We also observed that several crystals clumped together so they were not suitable for analysis. Regardless of these concerns we selected 1961 crystals for automatic diffraction and out of these only 913 (47%) delivered sufficiently good data for identification using unit cell parameters as discriminators. Since we also recorded the images of the crystals, we could go back and look at the morphologies of the crystals that did not diffract. Indeed, most of the problematic crystals had the issues described above suggesting that sample preparation should be improved in the future (Figure S1, Supporting Information). Despite this concern we could identify all components in the mixture and even identify the constituent with lowest percentage. Calcium acetate monohydrate which was added at ≈4.7% in the input was identified in 40 crystals corresponding to a 4.4% counting ratio (Figure S2, Supporting Information).

In the second set of mixed compounds, a total of 9 different saccharides, along with a similar scaffold (L‐Ascorbic acid), were weighed in with relative amounts ranging from 3.0 to 27.5% (Mixture B). The components of this mixture were: D‐Glucose (4.2% *v/v*), D‐Sucrose (3% *v/v*), D‐Maltose monohydrate (10.3% *v/v*), L‐Arabinose (4.2% *v/v*), L‐Ascorbic acid (13.1% *v/v*), D‐Galactose (6% *v/v*), D‐Trehalose dihydrate (19.7% *v/v*), D‐Xylose (11.9% *v/v*) and L‐Rhamnose monohydrate (27.5% *v/v*) (Table S4, Supporting Information). Separating some of the saccharides using size or affinity chromatography based on hydrophobicity or charge is challenging due to their chemical similarities. For instance, four of the saccharides, L‐Arabinose, D‐Xylose, D‐Galactose, and D‐Glucose, are pairwise chemically identical and differ only in the position of a hydroxyl group (diastereomers), which requires stereochemical separation. Trehalose and Maltose are also chemically identical, consisting of two glucose entities and differing only at the glycosidic linkage sites of the two pyranose rings. Similarly, sucrose is only one methylene group larger than maltose, which is similar in size. However, the crystallographic unit cells for all of these compounds are distinct from each other and easily distinguished in MicroED, making crystallographic analysis of chemically identical but stereochemically different compounds possible.

**Figure 2:**
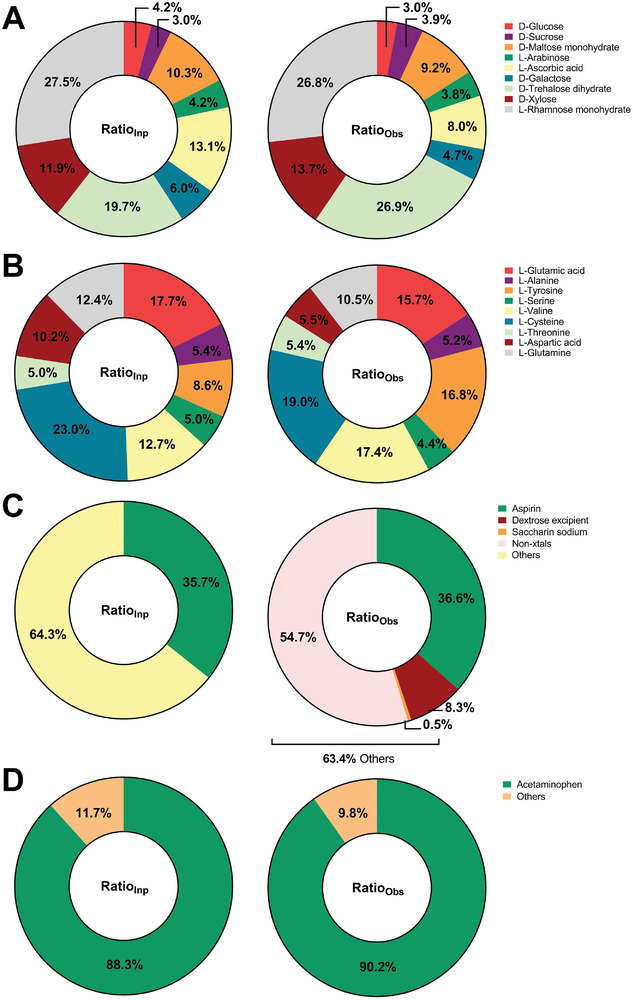
Compositional analysis. Compositional analysis of A) mixture B; B) mixture C; C) aspirin tablet; and D) acetaminophen tablet shows the relative volumes of the compounds in the actual mixture compared to the analysis based on automatically collected MicroED data. See Tables S4–S7 (Supporting Information).

For the saccharides mixture (B), we collected 1374 data sets automatically, of which 1000 (73%) were successfully identified as having one of the unit cells in the mixture. This was a substantial increase in success rate from 47% in mixture A to 73% in mixture B. As with Mixture A, the remaining 27% either did not diffract well possibly due to hydration, or originated from crystal clumps or were not crystalline to begin with. Importantly, all components of the mixture could be identified and for most the composition was determined within a 2% error. Only two compounds, L‐Ascorbic acid, and D‐Trehalose dihydrate, demonstrated larger errors of 5.1% and 7.2%, respectively. Although the reason for these discrepancies is not known, it was noted in other work that occasionally crystals have a tendency to lose their diffraction properties upon grinding.^[^ ^18^ ^]^ Deviations in the ratios of the constituents of the mixtures analyzed may come from differences in the size distributions of the sample crystals after grinding, and damages to the crystals during sample preparation. It is also likely that materials adhere differently to the grid, and this could also lead to minor errors. Since only the area is estimated and not the volume of the crystalline grains (see Experimental Section), this could be a potential source of uncertainty. It was noted however that the difference between the crystal counting results and the area‐corrected is small and the area estimation did not substantially influence the result (<2%). Overall, using MicroED in an automatic setting, only one grid containing the mixture was prepared and mounted, from which after processing the relative ratios could be derived (**Figure** **2**A; Table S6, Supporting Information).

We next applied the approach to a third class of compounds – amino acids. Mixture C contained L‐Glutamic acid (17.7% v/v), L‐Alanine (5.4% v/v), L‐Tyrosine (8.6% v/v), L‐Serine (5% v/v), L‐Valine (12.7% v/v), L‐Cysteine (23% v/v), L‐Threonine (5% v/v), L‐Aspartic acid (10.2% v/v), L‐Glutamine (12.4% v/v) (Table S4, Supporting Information). We selected 1401 crystals for automatic MicroED analyses and this time the success rate was even higher than for Mixture B as 1121 out of 1401 (80%) crystals were identified. The area‐corrected ratios (see Methods) of compounds in mixture C were found to be similar to the counting ratios obtained by simply counting the number of crystals, and with most ratios within 2% in relative amounts. However, for L‐Tyrosine, L‐Aspartic acid, and L‐Glutamine, which had larger relative errors, the area‐corrected ratios tended to be better than the counting ratios, particularly for L‐Tyrosine, where the overestimated ratio dropped from 10.6% to 8.2% after area correction. The estimated ratios are surprisingly close to the weighed‐in ratios in this analysis (Figure 2B; Table S6, Supporting Information). These results suggest that sample preparation and the material used for grid preparation are the key determinants of success. It is also possible that the amorphous carbon support used in these experiments is more suitable for biological material like amino acids.

As a further assessment of the general applicability of the method we applied the workflow on two commercially available drug tablets, formulations of Aspirin and Acetaminophen. Drug formulations may contain a variety of non‐active agents such as binders, disintegrants, sugar, and wax. They may not be in a crystalline state and could therefore potentially be problematic for a method based on the crystallinity of the compounds. In fact, the Aspirin formulation contains only 35.7% (w/w %) of the active ingredient in a formulation of mostly non‐active components. A few of the non‐active components were crystalline and their unit cell dimensions were found in the analysis. A substantial part however consists of ingredients for which the crystalline status is undeclared, such as for corn starch, carnauba wax, FD&C yellow no.6 aluminum lake and flavors. For starch alone, a partial crystallinity is often reported as well as several crystalline forms, making a complete analysis of this part of the formulation beyond our aim for this study. In order to extract valid ratios therefore, the number of crystals with a unit cell corresponding to the active ingredient were compared to the total amount of well diffracting as well as non‐diffracting grains selected. The remaining smaller group for which some diffraction was recorded but the diffraction was not enough for unit cell determination was considered as of undefined status and left out of the calculations as a whole. This was necessary as we could not conclude whether a weakly diffracting grain should be considered originating from a partially disordered crystalline grain or from an anticipated but partially organized amorphous phase. If such a grain originates from an anticipated amorphous phase, which is to some extent crystalline but not enough for determining the unit cell, for instance, the crystalline portion in our study could be overestimated. The analysis for Aspirin therefore, resulted in 36.6% of the diffracting crystals belonging to the unit cell of Aspirin and smaller fractions, 8.3% and 0.5% was identified as dextrose and saccharin respectively (Figure 2C; Table S7, Supporting Information). Considering the large amount of non‐diffracting grains, the error of less than one percent seems surprisingly accurate (Table S5, Supporting Information). For Acetaminophen on the other hand, the largest portion in the formulation, 88.3% (w/w %, Table S5, Supporting Information), consists of the active ingredient. A similar analysis was done for this sample which resulted in an estimated ratio of 90.2% of the active ingredient (Figure 2D; Table S7, Supporting Information). No other crystalline grains were found within this formulation.

**Figure 3:**
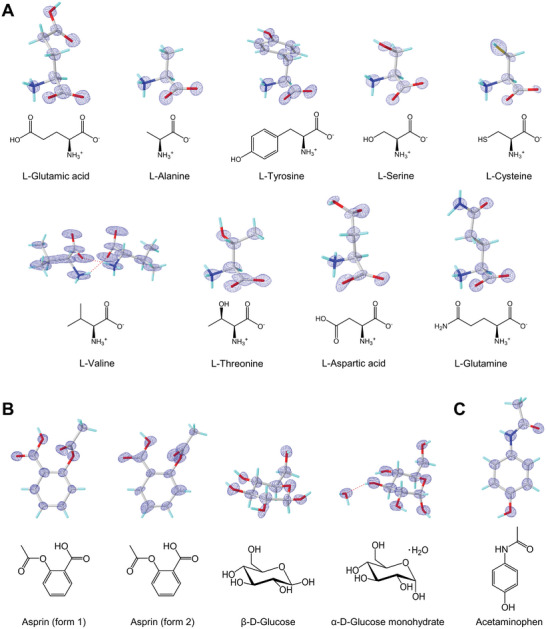
MicroED structures. The MicroED structures of A) nine amino acids in mixture C; B) aspirin (form 1 and 2), β‐D‐Glucose, and α‐D‐Glucose monohydrate in aspirin tablet; C) acetaminophen in acetaminophen tablet. Structures were solved by SHELXT30 and refined using SHELXL31 at 0.75 Å resolution. The blue meshes are 2Fo‐Fc electrostatic potential maps (presented by Olex2).^[^ ^34^ ^]^ The electrostatic potential level for each compound in 2Fo‐Fc are as listed: L‐Glutamic acid, 0.84 e·Å‐3; L‐Alanine, 0.80 e·Å‐3; L‐Tyrosine, 0.71 e·Å‐3; L‐Serine, 0.69 e·Å‐3; L‐Valine, 0.40 e·Å‐3; L‐Cysteine, 0.79 e·Å‐3; L‐Threonine, 0.74 e·Å‐3; L‐Aspartic acid, 0.37 e·Å‐3; L‐Glutamine, 0.52 e·Å‐3; aspirin (form 1), 0.74e·Å‐3; aspirin (form 2), 1.31 e·Å‐3; β‐D‐Glucose, 1.10 e·Å‐3; α‐D‐Glucose monohydrate, 1.21 e·Å‐3; acetaminophen, 1.04 e·Å‐3.

Although only a small wedge of data is collected from each crystal, data sets can be merged and structures solved as needed. We applied this approach to data sets from mixture C, aspirin, and acetaminophen tablets, and were able to solve all components to subatomic resolution (**Figure** **3**; Table S8, Supporting Information), with completeness levels greater than 80% and more than 2000 observed reflections in most cases even for these small molecules.

## 3 Conclusion

Methods such as chromatography and mass spectrometry^[^ ^7^, ^8^ ^]^ separate molecules based on difference in their sizes or chemical properties such as charge or hydrophobicity. For structures similar in size and chemical properties, like with isomers, separation is difficult. For crystalline samples the unit cell of a compound is a result of the exact packing of the molecules in order to lower the solid‐state energy and is a stereoselective 3D process^[^ ^11^ ^]^ Therefore, chemically similar compounds usually result in a very different crystal packing and sometimes also a different symmetry between the molecules, resulting in differences in space groups and unit cell parameters. As these can be determined rather accurately from processing the diffraction movies, the cells can easily be distinguished. Should the unit cell parameters accidentally be very close to each other for any two crystals, as a next step the difference between the individual intensities of two data sets can be used to discriminate between two structures, as understood from the fact that the intensity of a reflection hkl set can vary substantially with only a minor difference in the unit cell content.^[^ ^24^ ^]^ MicroED, just like PXRD, offers a way to analyze samples unrelated to chemical properties but rather from how they organize the molecules into a crystal.

Using PXRD for analytical purposes of samples complicated by several compounds or phases, diffraction patterns need to be known in advance for all the constituents using PXRD compositional analysis. The content of a mixture with crystalline constituents can be quantitatively analyzed with PXRD using the Reference Intensity Ratio (RIR). Typically it includes preparing a mixture for each of the components with an equal amount of a reference components from which the relative peak heights can be used as a reference. When the samples of unknown ratios are mixed with the reference component the searched for ratios can be estimated. Alternatively the peak heights can be calculated knowing the constituents of the unit cell. This is however limited from the assumption that the sample is 100% crystalline. In reality the crystalline phase of a compound may vary and it is only the crystalline portion of the sample that can be analyzed with a diffraction based technique such as PXRD and MicroED.

A neat feature with MicroED is that there is no need to know the unit cells in advance. It allows for precise estimates of the unit cell parameters, either from the individual data sets or after merging of several data sets, as long as the rotation per crystal allows a correct indexing of the diffraction pattern. In contrast, the successful merging of a few data sets typically allows the determination of the MicroED structure so that a more complete analysis of the sample is achieved. Therefore, if the content of the unit cell as well as the dimensions of the unit cell is known, there is no need to prepare a standard with previously known peaks and the diffraction patterns and the structures needed can instead be determined on the fly. The ability to use single nano crystals in MicroED and record the diffraction of a powder grain by grain, resolves peak overlap due to several interfering diffraction patterns and brings diffraction on powder samples into a new level of accuracy, only recently started to be explored. MicroED analysis of individual crystals can therefore be a powerful technique for complex samples with many constituents or otherwise overlapping powder diffraction patterns, yet with the same advantages as PXRD for identifying compounds in contrast to from their chemical profiles. It is our hope this will extend the use of diffraction based techniques for analysis of samples that are difficult to analyze from methods using hydrophobicity, charge, or size as discriminating factors.

If the content of the unit cell is not known in advance and at the same time not being determined due to limited diffraction or other limitations, the size of the particles can be estimated and with a density of the samples the weight fraction can be calculated. In this study we measured the visible area of each crystal in order to estimate its volume, and final result is to some extent dependent on the size estimation of the diffracting crystals. However, even without a reliable way to estimate the diffracting volume properly as the thickness of the sample was not estimated, the results obtained from just counting the number of crystals are similar to the result where the area for each crystal is considered as well. As an example, the differences between the counted ratios and the area corrected ratios for each compound in sample B and C, is considerably smaller than the actual errors of the same ratios (σ is 0.95% and 3.5% respectively). We therefore conclude that we could achieve close to the final result also without a careful volume estimation selecting crystals for analysis within a suitable size range in the selection process. As other errors, such as density calculation of the known ratios and substance handling errors, tend to be substantially less, our interpretation is that the largest error is related to the diffraction properties of the individual crystals and the crystallinity of the compounds. It is commonly accepted that also for PXRD analytical purposes, a 100% crystallinity can not be achieved and a detection limit of a few percent has been recorded for applications like PXRD, DSC, and Raman.^[^ ^36^ ^]^

We compared our results with the latest round Robin assessment that was performed for PXRD in 2009.^[^ ^35^ ^]^ In that assessment Mannitol, Acetaminophen, and Silicon were used as a mixture containing two or three components at various amounts. While this test is much simpler in composition that our tests using MicroED we note that with the PXRD tests the errors in compositional assessment were rather large, between 9 and 11% while with MicroED, even though the samples were much more complex the errors currently hover around a mere 2–3%. While the PXRD tests are more than a decade old and the technology may have advanced, this comparison suggests to us that MicroED should be considered as a complementary, or alternative, to PXRD in future studies.

When the data collection and analysis, which can be time consuming for a large number of data sets, the automation of MicroED offers several advantages. In addition to the increased resolution and automatic structure determination, it is possible to retrieve the structures of the ingredients of a mixture even for compounds in very small ratios, whereas in PXRD a structural analysis is substantially aided by an homogenous sample containing a single phase. Also, using MicroED there is no limit as to how many compounds that can be separated from a mixture, whereas overlap of the diffraction rings easily may limit the number of diffraction patterns that can be separated in PXRD.

However, further improvements in sample preparation^[^ ^14^ ^]^ are necessary to fully exploit the potential of MicroED for analytical purposes. The experiments conducted using the three mixtures have provided us with valuable insights into the factors that may impact compositional analysis. It was observed during sample preparation that the crystals had different physical properties, which influenced the efficiency of grid preparation. For instance, some materials were harder and less prone to breaking into the required size for MicroED, while others were brittle and more easily prepared. Second, electrostatic charging of the powder during grinding can cause an excess of powder to attach to the interior of the vial rather than onto the EM grid. Third, the physical shearing of the sample may impact the crystallinity, although this is more often experienced with protein samples rather than small molecules or salts. For a few compounds we observed behaviors that we interpreted as them being hygroscopic. Some crystals of one compound appeared to be in a partially “melted” phase and we observed that some crystals clumped together more than otherwise seen so they were not suitable for analysis. The carboxylic group of citric acid is slightly hygroscopic (can attract the equivalent of one water per molecule in equilibrium in), while D‐Trehalose already contains crystalline water, whereas many sugar compounds are less hygroscopic. We suggest subsequent work on hygroscopic nano crystals should be carried out in a humidity controlled environment, as is often the case for laboratories connected to electron microscope sample preparation for CryoEM.

For some crystals the diffraction was recorded but the diffraction was not enough for unit cell determination. These were considered undefined and left out of the calculations as a whole. This diffraction could be mostly amorphous material that is partially more structured, in which case leaving them out of the calculation would be the consistent way to proceed. This situation is no different than any case where the crystalline portion is not close to 100%. It can also be the case that a few crystals were too small or there were several crystals with interfering diffraction, which then would underestimate the results for all compounds where this holds true. We suggest that with more experience in automatic data collection there will be a more optimized way to select targets that are more appropriate for diffraction analysis, avoiding the crystals that are visually problematic.

Notably, we observed that 70–80% of the selected crystals underwent automatic data processing and were successfully identified. We could conclude that a few crystals were too small to produce a high‐quality diffraction pattern or were damaged prior to the experiment. We further noticed that the carbon support was not always flat even within a single grid square which could have led to errors in the estimation of the eucentric height of individual crystals. As a result, a few crystals partially rotated out of the beam center during data collection. It is likely that with improved crystal selection criteria and a local eucentric height determination, the throughput could be further improved to the limitations of the sample itself, determined by variations in the diffraction properties of individual crystals within the sample.

These advantages of using MicroED for analytical purposes are not without considerations though, the most obvious being the cost of the experiment. In comparison to PXRD which is the commonly used and straightforward method for mixtures with non‐overlapping peaks and already available standards measurements, the electron microscopy time is substantially more expensive. Collecting data from thousands of crystals currently requires a day even in this automatic setup and is followed by the processing of each data set. The cost should decrease as dedicated MicroED systems are developed.

The main purpose of the data collection here was to analyze the constituents rather than to solve the structures, which is why a short rotation range of only 50° per crystal was used instead of the typical larger rotation range that would be more appropriate for structure determination. This decision facilitated the collection of more data sets within the same time frame, increasing the statistical significance of the analysis. Attempts to use much shorter rotation wedges underlined the difficulties in indexing the data sets reliably due to the flatness of the Ewald's sphere. As an example at the extreme end, no rotation was used in another study where instead prior knowledge of the unit cell parameters was used to compensate for the lack of rotation data.^[^ ^26^ ^]^ Our approach involved continuous rotation of at least 20° per crystal and allows unit cell determination without any a priori knowledge,^[^ ^1^, ^2^ ^]^ which is more suitable for analytical purposes. The standard deviations of the determined unit cell parameters are well within the tolerances (a maximum 1Å deviation in length and 10° deviation in angles) used and the compounds with a correctly determined unit cell could be unambiguously assigned (see Figures S3–S6, Tables S9 and S10, Supporting Information). All the determined unit cells showed high degree of correlation to the reference values (Figures S7–S10, Supporting Information). Our study also demonstrates that it is not necessary to solve the structures for compositional analysis, as proper unit cell parameters are sufficient to distinguish between the compounds in the mixtures. This approach also allows for a more comprehensive analysis of the sample when the structures of the constituents are already known, as indexing a data set is typically easier than solving the structure ab initio.

Recent trends in structural biology emphasize the importance of collecting multiple sets of redundant data, especially for systems with low signal‐to‐noise ratios. This has significant implications for MicroED. First, for complex systems where only a fraction of a sample diffracts to the desired resolution, a major part of the data collection process is dedicated to finding the best crystals. Second, redundant data can improve the signal‐to‐noise ratio and reduce the impact of systematic artifacts during data collection.^[^ ^27^ ^]^ Finally, merging datasets to increase completeness is often necessary, but the outcome of this approach depends on the isomorphism of the data sets.^[^ ^28^ ^]^ In particular, since phasing using ab initio methods depends entirely on the structure factors, reliable estimates are required. A large pool of data sets facilitates the prospect of finding isomorphous data sets that could then be merged together to produce a complete data set for structure determination. This approach was demonstrated here with Mixture C where despite the use of a short rotation range, all structures in mixture C were successfully solved as a step toward future high throughput MicroED structure determination. The sample preparation, which merely consists of grinding the sample to a fine homogeneous powder, mixing the powder with an electron microscopy grid, and subsequently loading the grid in the microscope is typically done in minutes and has been described elsewhere. The setting up of the low magnification atlas and the medium magnification montages is straightforward and requires 15 min of human intervention and 2–8 h of automatic data collection in image mode, depending on the number of tiles of the medium magnification montage requested. The crystal selection in this study was done manually with another 2–4 man‐hours which initiated the automatic data collection of ≈750 crystals in, accounting for the remaining 15 h of a 24‐h shift of the microscope. The script, which automatically processes each of the data sets and merges combinations of the data sets collected, is finished within 12–72 h depending on the settings and the hardware used. The result of the composition analysis and the processing is summarized in a few text files. Thus, the whole process typically takes 2 days on 750 data sets and requires a few hours of manual work mostly for the crystal selection process.

This study presents an automatic approach to MicroED using commonly used and freely available software. The data collection process requires no human intervention after initial setup, making it suitable to run during less busy microscope shifts. This approach is built on the widely distributed CryoEM data collection software, SerialEM, which is already installed at many CryoEM labs. The hope is that by using commonly available software, this will inspire more laboratories to implement a higher level of automation in their MicroED data collection processes. In summary, the high throughput automatic MicroED approach established and demonstrated in this study has great potential for expanding the applications of MicroED as an analytical tool well beyond a structural determination tool. With the ability to collect and analyze vast amounts of data, MicroED can be used for compositional analysis, providing a reliable and statistically significant analysis of the relative composition of a sample.

## 4 Experimental Section

### Materials

All the compounds were commercially purchased and used as received without further recrystallization. D‐Glucose, D‐Sucrose were purchased from Acros Organics. L‐Valine was purchased from Alfa Aesar. Sodium bicarbonate, Magnesium sulfate heptahydrate, Sodium citrate dihydrate, Calcium acetate monohydrate, D‐Galactose, L‐Ascorbic acid were purchased from Fisher Chemical. Sodium sulfate, Potassium sulfate, Sodium chloride, Calcium gluconate, Magnesium acetate tetrahydrate, D‐Maltose monohydrate, D‐Trehalose dihydrate, L‐Alanine, L‐Arabinose, L‐Aspartic acid, L‐Cysteine, L‐Glutamic acid, L‐Glutamine, L‐Rhamnose monohydrate, L‐Serine, L‐Threonine, L‐Tyrosine were purchased from Sigma‐Aldrich. D‐Xylose was purchased from Tokyo Chemical Industry (TCI). The Aspirin tablets (NDC: 59779‐467‐68) and Acetaminophen tablets (NDC: 69842‐484‐62) were purchased from CVS pharmacy.

### Sample Preparation

Compounds in Mixture A‐C were carefully weighed by a Mettler Toledo (XPR225DR) analytical balance and mixed in a 20 mL scintillation vial. Mixture A was prepared as follows: Sodium bicarbonate (16.32 mg), Sodium sulfate (29.31 mg), Potassium sulfate (19.49 mg), Magnesium sulfate heptahydrate (9.14 mg), Sodium chloride (6.07 mg), Calcium gluconate (5.14 mg), Sodium citrate dihydrate (13.92 mg), Calcium acetate monohydrate (3.66 mg), Magnesium acetate tetrahydrate (8.93 mg). Mixture B was prepared as follows: D‐Glucose (5.31 mg), D‐Sucrose (3.88 mg), D‐Maltose monohydrate (14.7 mg), L‐Arabinose (5.42 mg), L‐Ascorbic acid (17.55 mg), D‐Galactose (7.3 mg), D‐Trehalose dihydrate (28.05 mg), D‐Xylose (14.73 mg), L‐Rhamnose monohydrate (32.73 mg). Mixture C was prepared as follows: L‐Glutamic acid (22.72 mg), L‐Alanine (6.45 mg), L‐Tyrosine (10.47 mg), L‐Serine (6.62 mg), L‐Valine (13.04 mg), L‐Cysteine (24.95 mg), L‐Threonine (5.5 mg), L‐Aspartic acid (14.11 mg), L‐Glutamine (14.13 mg) (Tables S3 and S4, Supporting Information). The volume percent of each compound was calculated to generate a wide ratio range, from 3.0 to 27.5%. The compounds in the respective mixture were mixed together before being ground separately by an agate mortar and pestle set (internal diameter 50 mm) three times at room temperature to yield a fine powder without any visible crystalline solids left. The total weight of each mixture was more than 100 mg to ensure a thorough interaction with the agate mortar and pestle during the grinding process. One aspirin tablet (227.17 mg) and one acetaminophen tablet (566.26 mg) were weighed by Mettler Toledo (XPR225DR) analytical balance separately, and ground by an agate mortar and pestle set three times to yield the fine powders (same as mixture A‐C).

### Grid Preparation

The carbon‐coated copper grids (400‐mesh, 3.05 mm O.D., Ted Pella Inc.) were pretreated with glow‐discharge plasma at 15 mA on the negative mode using PELCO easiGlow (Ted Pella Inc.), with no glow discharge for mixture A, 60 s glow discharge time for mixture B, aspirin tablet, and acetaminophen tablet, and 30s for mixture C. Around 1 mg powder from each set was transferred to a 10 mL scintillation vial and separately mixed with the grid. After a gentle shaking of the vial, the grids were taken out and clipped at room temperature.

### Automatic MicroED Data Collection with SerialEM

The clipped grids were loaded in an aligned Thermo Fisher Talos Arctica Cryo‐TEM (200 kV, ≈0.0251 Å) at 100 K, equipped with a Falcon III direct electron detector (4096 × 4096 pixels). Intensity of 45.2% was found to be the condition for parallel beam during diffraction using the contrast of the objective aperture.^[^ ^29^ ^]^ For diffraction movies the data was collected with an 829 mm diffraction length and using the smallest C2 aperture of 20 µm without the selected area aperture. The resulting beam size was ≈1.5 µm. MicroED data was automatically collected using the SerialEM software in microprobe mode.

An atlas over the entire grid was collected as a low‐magnification montage of 8 × 8 tiles. In the next step, typically more than 150 grid squares of interest were selected, and points were saved using “Add Points” in the SerialEM navigator window. After aligning the atlas with the magnification used in the medium magnification montages, one medium magnification montage of 3×3 pieces was collected at all saved points using the View mode. The “Rough eucentricity” and “Fine eucentricity” functions in SerialEM were used to automatically assign the eucentric height to each grid square that was stored with the corresponding maps. A point was added for each crystal of interest in the medium montage maps and saved in the navigator window. All points in the generated list were set up for data collection using the command “Acquire at items” function in the Navigator menu of SerialEM. TEM based techniques require samples thin enough to allow penetration of the electron beam and the purpose of selecting crystals in this size range only was to increase the chance of selecting crystals thin enough for an interpretable diffraction pattern. Care was taken during sample preparation to ensure a uniform grinding of the sample to achieve a homogeneous size distribution. Crystals ranging from 0.2 to 1.5 µm (size of the parallel beam) were picked for the data collection. Typical diffraction data using continuous rotation of the stage at 2°/s covering a total rotation range from −25° to +25° (−40° to +40° for aspirin and acetaminophen tablets) was automatically collected for each crystal using a SerialEM macro script. The script also used image mode to save an image of the content in the beam using the Search preset and was used to estimate crystal area and to visualize the crystal position during the MicroED data acquisition (Figure 1). During the rotation, the camera integrated frames continuously at a rate of ≈0.5s per frame, a total of 24.95s exposure time for 50 frames (39.92s for 80 frames).

### Automatic Processing

An in‐house developed python script automatically processed the MicroED data via three steps using available software: (1) the raw MicroED data in MRC format were automatically converted to SMV format using mrc2smv software (https://cryoem.ucla.edu/downloads/snapshots);^[^ ^22^ ^]^ (2) the converted data were processed in XDS^[^ ^23^ ^]^ and (3) data sets were merged using XSCALE.^[^ ^24^ ^]^ XDS used a few typical settings in input: the detector distance is not refined together with the unit cell refinement due to the flatness of the Ewald's sphere, DELPHI was set to 30°, maximum errors were set to 10 and 3 for spot and spindle respectively, and occasionally we used MINIMUM_FRACTION_OF_INDEXED_SPOTS equals 0.1 to include weak data. (See “XDS.INP template”, Supporting Information). The script evaluates different settings in XDS input for STRONG_PIXEL, SIGNAL_PIXEL, MINIMUM_NUMBER_OF_PIXELS_IN_A_SPOT, OFFSET, DATA_RANGE, SPOT_RANGE. The best merged data was found by evaluating all combinations of data sets to a certain maximal number of data sets included and the solutions were scored using statistics from XSCALE. The structures were solved using SHELXT^[^ ^30^ ^]^ and refined with SHELXL.^[^ ^31^ ^]^

### Compositional Analysis and Crystal Area Estimation

For the mixtures with the known crystal structure, the literature‐reported unit cells in Cambridge Structure Database (CSD) were used as a reference for the identification of a compound, as well as to phase data for the structure determination. The crystals were grouped according to compounds from the diffraction data and the relative ratios were calculated for each compound based on the number of dataset for each compound, which we refer to as the “counting ratio”. For the assignment of the proper crystal structure to an indexed data set, a maximum 1Å length tolerance and 10° angle tolerance were used (see Figures S3–S6, Tables S9 and S10, Supporting Information). These limits ensured that crystals were appropriately identified by the unit cells within reasonable error. The pattern of the unit cell parameters was unique for every crystal form and with no overlap between any two unit cells and could therefore be used to unambiguously assign the proper crystal structure to any indexed data set. For each sample a certain percentage of the data sets could not be assigned to a particular unit cell. This group contains images with no diffraction, smeared diffraction, multiple lattices, or too low resolution for the software to be able to recognize the diffraction pattern. In case of no diffraction, the images recorded in parallel often showed that the beam had not hit the crystal properly.

The search images as saved in MRC format were converted to TIFF format using mrc2tif software.^[^ ^32^ ^]^ Then the converted images were imported into ImageJ software^[^ ^33^ ^]^ with the Threshold values to be adjusted until the whole crystals were fully detected (colored in red, Figure 1). In ImageJ, the “Analyze Particles” function was used to automatically count the number of pixels corresponding to the crystal, (colored in black, Figure 1) and area was manually inspected to ensure to only include the crystal area. The crystal area was calculated and summed for each component (*S_Comp_*) and for each composition (*S_Total_*)for ratio analysis.

A second ratio of a compound referred to as the “area‐corrected ratio” and Ratio_Obs_ in Figures 2, S6 and S7 (Supporting Information) and was calculated by the Equation (1) as shown below:

(1) $${\mathrm{Ratio}}_{\mathrm{Obs}}=\frac{S_{\mathrm{Comp}}}{S_{\mathrm{Total}}}$$

The ratio of the total area of one component (*S_Comp_*) to the total area of all crystals (*S_Total_*) for successfully indexed data sets only. The input ratio and observed ratio were plotted by Graphpad Prism 8.0.1 for Windows (GraphPad Software, San Diego, California USA, www.graphpad.com) as shown in Figure 2.

## Conflict of Interest

The authors declare no conflict of interest.

## Author Contributions

J.U. and J.L contributed equally. J.U. designed the experiments, developed the sample preparation techniques and the data collection workflow, wrote the Python script for automatic processing, participated in data analysis, managed the activities, and assisted in manuscript preparation. J.L. performed the sample preparation, performed the data collection, analyzed the data and prepared the tables, performed the refinement and structure determination, prepared the figures, and assisted in manuscript preparation. S.W. prepared the initial setup of the microscope workflow and data processing. A.S. provided experience in sample preparation, data analysis, and microscope setup. T.G. conceived of the project, designed experiments, provided expertise, assisted with manuscript preparation, and supervised the project.

## Supporting information

Supporting Information

## Data Availability

The data that support the findings of this study are available from the corresponding author upon reasonable request.

## References

[1] D. Shi , B. L. Nannenga , M. G. Iadanza , T. Gonen , elife. 2013, 2, e01345. 24252878 10.7554/eLife.01345 PMC3831942

[2] B. L. Nannenga , D. Shi , A. G. W. Leslie , T. Gonen , Nat. Methods. 2014, 11, 927. 25086503 10.1038/nmeth.3043 PMC4149488

[3] M. W. Martynowycz , M. T. B. Clabbers , J. Unge , J. Hattne , T. Gonen , Proc. Natl. Acad. Sci. USA 2021, 118, e2108884118. 34873060 10.1073/pnas.2108884118 PMC8670461

[4] H. N. Chapman , Protein Crystallography: Methods and Protocols. 2017, 1607, 295.

[5] K. Diederichs , M. Wang , Protein Crystallography: Methods and Protocols. 2017, 1607, 239. 10.1007/978-1-4939-7000-1_10 28573576

[6] R. Henderson , Q. Rev. Biophys. 1995, 28, 171. 7568675 10.1017/s003358350000305x

[7] K. J. Kumar , V. Vijayan , Pharmaceutical methods. 2014, 5, 47.

[8] R. H. Rathod , S. R. Chaudhari , A. S. Patil , A. A. Shirkhedkar , Future J. Pharm. Sci. 2019, 5, 1.

[9] X. Li , K. Hu , Annu. Rep. NMR Spectrosc. 2017, 90, 85.

[10] C. F. Holder , R. E. Schaak , ACS Nano. 2019, 13, 7359. 31336433 10.1021/acsnano.9b05157

[11] A. Abelian , M. Dybek , J. Wallach , B. Gaye , A. Adejare , The Science and Practice of Pharmacy, , In Remington 2021 pp. 105.

[12] B. Wang , X. Zou , S. Smeets , IUCrJ. 2019, 6, 854. 31576219 10.1107/S2052252519007681 PMC6760450

[13] S. Smeets , X. Zou , W. Wan , J. Appl. Crystallogr. 2018, 51, 1262. 30279637 10.1107/S1600576718009500 PMC6157704

[14] C. G. Jones , M. W. Martynowycz , J. Hattne , T. J. Fulton , B. M. Stoltz , J. A. Rodriguez , H. M. Nelson , T. Gonen , ACS Cent. Sci. 2018, 4, 1587. 30555912 10.1021/acscentsci.8b00760 PMC6276044

[15] M. Ge , Y. Wang , F. Carraro , W. Liang , M. Roostaeinia , S. Siahrostami , D. M. Proserpio , C. Doonan , P. Falcaro , H. Zheng , Angew. Chem., Int. Ed. 2021, 133, 11492. 10.1002/anie.202016882 PMC8252586 33682282

[16] E. T. Broadhurst , H. Xu , S. Parsons , F. Nudelman , IUCrJ. 2021, 8, 860. 34804540 10.1107/S2052252521010101 PMC8562671

[17] Y. Luo , B. Wang , S. Smeets , J. Sun , W. Yang , X. Zou , Nat. Chem. 2023, 15, 1384. 36717616 10.1038/s41557-022-01131-8 PMC10070184

[18] T. Sasaki , T. Nakane , A. Kawamoto , T. Nishizawa , G. Kurisu , CrystEngComm. 2023, 25, 352.

[19] D. N. Mastronarde , J. Struct. Biol. 2005, 152, 36. 16182563 10.1016/j.jsb.2005.07.007

[20] M. Schorb , I. Haberbosch , W. J. H. Hagen , Y. Schwab , D. N. Mastronarde , Nat. Methods. 2019, 16, 471. 31086343 10.1038/s41592-019-0396-9 PMC7000238

[21] M. J. de la Cruz , M. W. Martynowycz , J. Hattne , T. Gonen , Ultramicroscopy. 2019, 201, 77. 30986656 10.1016/j.ultramic.2019.03.009 PMC6752703

[22] J. Hattne , M. W. Martynowycz , P. A. Penczek , T. Gonen , IUCrJ. 2019, 6, 921. 31576224 10.1107/S2052252519010583 PMC6760445

[23] W. Kabsch , Acta Crystallogr. D. 2010, 66, 125. 20124692 10.1107/S0907444909047337 PMC2815665

[24] W. Kabsch , Acta Crystallogr. D. 2010, 66, 133. 20124693 10.1107/S0907444909047374 PMC2815666

[25] F. H. C. Crick , B. S. Magdoff , Acta Crystallogr. 1956, 9, 901.

[26] R. Bücker , P. Hogan‐Lamarre , P. Mehrabi , E. C. Schulz , L. A. Bultema , Y. Gevorkov , W. Brehm , O. Yefanov , D. Oberthür , G. H. Kassier , Nat. Commun. 2020, 11, 996. 32081905 10.1038/s41467-020-14793-0 PMC7035385

[27] P. A. Karplus , K. Diederichs , Curr. Opin. Struct. Biol. 2015, 34, 60. 26209821 10.1016/j.sbi.2015.07.003 PMC4684713

[28] J. Foadi , P. Aller , Y. Alguel , A. Cameron , D. Axford , R. L. Owen , W. Armour , D. G. Waterman , S. Iwata , G. Evans , Acta Crystallogr. D. 2013, 69, 1617. 23897484 10.1107/S0907444913012274 PMC3727331

[29] M. A. Herzik , cryoEM: Methods and Protocols. 2021, 2215, 125. 10.1007/978-1-0716-0966-8_6 33368002

[30] G. M. Sheldrick , Acta Crystallogr. A. 2015, 71, 3.

[31] G. M. Sheldrick , Acta Crystallogr. C. 2015, 71, 3.

[32] J. R. Kremer , D. N. Mastronarde , J. R. McIntosh , J. Struct. Biol. 1996, 116, 71. 8742726 10.1006/jsbi.1996.0013

[33] C. A. Schneider , W. S. Rasband , K. W. Eliceiri , Nat. Methods. 2012, 9, 671. 22930834 10.1038/nmeth.2089 PMC5554542

[34] O. V. Dolomanov , L. J. Bourhis , R. J. Gildea , J. A. K. Howard , H. Puschmann , J. Appl. Crystallogr. 2009, 42, 339. 10.1107/S0021889811041161 PMC3236671 22199401

[35] T. G. Fawcett , F. Needham , J. Faber , C. E. Crowder , Powder Diffr. 2010, 25, 60.

[36] J. A. Newman , P. D. Schmitt , S. J. Toth , F. Deng , S. Zhang , G. J. Simpson , Anal. Chem. 2015, 87, 10950. 26465382 10.1021/acs.analchem.5b02758 PMC4747326

