# Supplementary material for: Compositional Analysis of Complex Mixtures using Automatic MicroED Data Collection
Source: Adv Sci (Weinh). 2024 Apr 22;11(23):2400081. doi: 10.1002/advs.202400081 (PMC11187898; doi:10.1002/advs.202400081)
Supplement: Supplementary file 1 — Supporting Information [file ADVS-11-2400081-s001.pdf]

ADVANCED SCIENCE
Open Access

Supporting Information

for *Adv. Sci.*, DOI 10.1002/advs.202400081

Compositional Analysis of Complex Mixtures using Automatic MicroED Data Collection

*Johan Unge, Jieye Lin, Sara J Weaver, Ampon Sae Her and Tamir Gonen**

Supporting information

Automatic MicroED Data Collection Enables Compositional Analysis

Johan Unge[1#], Jieye Lin[1#], Sara J Weaver[1], Ampon Sae Her[1] and Tamir Gonen[1,2,3]*

[1]Department of Biological Chemistry, University of California, Los Angeles, 615 Charles E. Young Drive South, Los Angeles, California 90095, United States

[2] Department of Physiology, University of California, Los Angeles, 615 Charles E. Young Drive South, Los Angeles, California 90095, United States

[3] Howard Hughes Medical Institute, University of California, Los Angeles, Los Angeles, California 90095, United States

Table of Contents

1. Additional Text

    Automatic high throughput MicroED Data Collection

    Using high throughput MicroED for compositional Analysis

    Data processing

2. Tables S1-S6

3. Figures S1-S3

1. Additional Text

Automatic high throughput MicroED Data Collection

Although automation often requires a significant amount of software development, the approach used in this work utilized widely available software, such as SerialEM and standard X-ray crystallographic processing software. The method involved collecting an atlas montage of the entire grid, followed by medium-magnification montages of the most promising grid squares (Figure 1 in the main article). The eucentric height was automatically determined for each grid square and stored with the medium montage maps. The crystal positions were accurately determined from the montage maps and stored as a list of points in SerialEM. Each point in the list was queued for data collection, applying a SerialEM macro to each point. Typically, each grid square contained 10 crystals of the appropriate size and thickness, and about 150 medium magnification montages were needed to assemble a data collection of 1500 crystals. A typical data set of 50° continuous rotation collected at 2°/s took approximately one minute to collect on the Talos Arctica and over 1000 complete data sets were collected automatically overnight.

We developed a Python (to be published) script to process data generated from the automatic approach in real time. This script was designed to keep up with the speed of data acquisition and to customize the processing for MicroED data recorded using the Falcon detector and the Talos Arctica. The pipeline includes converting the mrc image file format to smv format (using software that is freely available on our lab website https://cryoem.ucla.edu/microed), and running XDS, XSCALE, and XDSCONV to process, merge, and convert the data to SHELX hkl file format. The pipeline uses a brute force approach to evaluate a combination of frequently used input parameters as the best statistics are usually not found with the same XDS input parameters. The best solution is selected based on the results of XSCALE, and the pipeline systematically explores combinations of data sets to merge, and finally uses a scoring function to select the top merged data sets which are suggested to the user. This approach resulted in a robust pipeline to process MicroED data from various samples without the need to select input parameters prior to processing or assuming similarity among data sets.

Using high throughput MicroED for compositional Analysis

Once the data was collected and processed, we used the unit cell parameters for chemical identification (Table S6). Using the unit cell parameters to identify the crystal structures has several advantages over solving structures for all data sets. First, it allows for shorter rotation wedges to be collected for each data set, increasing the number of data sets that can be collected within a set time. Second, the unit cell parameters are easier to extract than solving structures for all data sets. This is particularly advantageous when collecting data on a large number of crystals that vary in size and diffraction quality, as some data sets may not be good enough for proper structure determination but sufficiently good for accurate unit cell and symmetry determination. Finally, using unit cell parameters to identify structures results in a higher accuracy of assignments, which is crucial for the identification of compounds present in small amounts in the mixture. Overall, this approach allowed for the successful identification of all salts, including those present in the lowest amounts in the mixture.

To validate the approach, we performed an analysis to assess the risk of incorrectly assigning a dataset to the wrong crystal structure based on similarities in the unit cell parameters. We established narrow windows around the published values for the unit cell dimensions and angles, allowing for deviations of up to 1Å and 10°, respectively. These restrictions were compared with

the actual distributions of unit cell parameters in the sample mixtures, and the results showed that the actual parameters fell well within the allowed values (Figures S1 and S2). Furthermore, we found no overlap in the absolute values and combinations of unit cell parameters for any two crystal forms, indicating that the proper crystal structure could be unambiguously determined for all the compounds involved in the experiments. Overall, the analysis confirmed that the unit cell parameters were unique and easy-to-compare properties for the crystal structures, and accurately identified the compounds in the mixtures.

In addition, to validate the crystal selection process, the diffraction quality of the selected crystals was analyzed. Moreover, the refined R_1 factors reflect an overall excellent MicroED data quality with no significant difference between the compounds. These results suggest that the crystal selection process did not introduce significant bias in the diffraction quality or thermal motion of the selected crystals. For each crystal, the volume of the diffracting part was estimated by multiplying the area of the crystal by a thickness factor that accounts for the variation in crystal thickness. This thickness factor was calibrated by comparing the electron transmission through several crystals of known thickness, and it was assumed to be constant for all crystals within the selected size range. The estimated crystal volumes were found to be similar across all compounds, indicating that the sample preparation process did not result in a bias towards a particular compound due to differences in crystal hardness or brittleness.

Data processing

To handle the thousands of datasets produced in these MicroED experiments, an automated approach for data analysis was necessary. In addition to the commonly used pipelines for X-ray structure determination, semi-automated MicroED focused systems have been published, but none are fully automatic. For this study a new Python script has been developed for an optimal treatment of MicroED data. The script runs all the steps from image conversion through integration to small molecule structure determination and at the same time optimizes input parameters iteratively. MicroED data requires slightly different input parameters compared to X-ray data due to the different setup. Typically, the X-ray beam generated by an insertion device at a synchrotron storage ring takes a rather long and straight path only bent by mirrors for the purposes of focusing the beam and is inherently parallel by the long distance between the source and the sample. In an electron microscope the electrons are easily bent by electronic lenses which are used to focus, shape, tilt and size, making the beam parallel as well as compensating for deviations introduced by previous lens imperfections. As a result, the exact position of a lattice point in the 3-dimensional reciprocal lattice, and therefore also its reflection onto the detector surface, is affected by the electron beam alignment to a larger extent than for X-ray or neutrons. Therefore, a more generous expectation in the errors of the lattice should be introduced to processing software like XDS.

2. Tables

	Mixture A	Mixture B	Mixture C
No. of grid squares montaged	34	164	92
No. of data sets (xtals) collected	1961	1374	1401
No. of xtals identified	913	1000	1121

Table S1 Statistics from the various steps of SerialEM data collection for Mixture A-C.

	Aspirin tablet	Acetaminophen tablet
No. of grid squares montaged	159	143
No. of data sets collected	1588	756
No. of unit cells identified	579	652
No. of non-crystalline data sets	715	56
Sets with undefined status	294	48

Table S2 Statistics from the various steps of SerialEM data collection for Aspirin tablet and Acetaminophen tablet. Sets of undefined status were typically diffraction patterns not good enough for unit cell determination. This group likely contains several components of the non-active ingredients with undeclared diffraction properties such as starch, carnauba wax, FD&C yellow no.6 aluminum lake and flavors. Starch alone is reported to have several crystalline states with various degrees of crystallinity and the group with sets of undefined status was therefore removed from evaluation as a whole.

Mixture A	Density (g/cm^3)	Mass (mg)	No. xtals
Sodium bicarbonate	2.20	16.32	19
Sodium sulfate	2.67	29.31	95
Potassium sulfate	2.66	19.49	122
Magnesium sulfate heptahydrate	2.66	9.14	19
Sodium chloride	2.17	6.07	5
Calcium gluconate	1.68	5.14	4
Sodium citrate dihydrate	1.76	13.92	136
Calcium acetate monohydrate	1.50	3.66	40
Magnesium acetate tetrahydrate	1.45	8.93	126

Table S3 Input composition and the number of crystals identified in mixture A.

Mixture B	Density (g/cm^3)	Mass (mg)	Volume (mm^3)	% *v/v*
D-Glucose	1.56	5.31	3.40	4.2
D-Sucrose	1.59	3.88	2.44	3.0
D-Maltose monohydrate	1.77	14.70	8.31	10.3
L-Arabinose	1.59	5.42	3.41	4.2
L-Ascorbic acid	1.65	17.55	10.64	13.1
D-Galactose	1.50	7.30	4.87	6.0
D-Trehalose dihydrate	1.76	28.05	15.94	19.7
D-Xylose	1.53	14.73	9.63	11.9
L-Rhamnose monohydrate	1.47	32.73	22.27	27.5
Mixture C	Density (g/cm^3)	Mass (mg)	Volume (mm^3)	% *v/v*
L-Glutamic acid	1.54	22.72	14.75	17.7
L-Alanine	1.43	6.45	4.51	5.4
L-Tyrosine	1.46	10.47	7.17	8.6
L-Serine	1.60	6.62	4.14	5.0
L-Valine	1.23	13.04	10.60	12.7
L-Cysteine	1.30	24.95	19.19	23.0
L-Threonine	1.31	5.50	4.20	5.0
L-Aspartic acid	1.66	14.11	8.50	10.2
L-Glutamine	1.36	14.13	10.39	12.4

Table S4 Input composition in mixture B and C.

Aspirin tablet[a,b]	Mass (mg)	% *w/w*
Aspirin	81	35.7
Others	146.17	64.3
Acetaminophen tablet[a,c,d]	Mass (mg)	% *w/w*
Acetaminophen	500	88.3
Others	66.26	11.7

Table S5 Ingredients composition in Aspirin and Acetaminophen tablet. [a]composition was obtained from the “Drug facts” declared on the packages. [b]Aspirin tablet contains 81 mg aspirin (active ingredient) and others (inactive ingredients) including corn starch, dextrose excipient, FD&C yellow no.6 aluminum lake, flavors, saccharin sodium. [c]Acetaminophen tablet contains 500 mg acetaminophen (active ingredient) and others (inactive ingredients) including carnauba wax, hypromellose, polyethylene glycol, povidone, pregelatinized starch, stearic acid. [d]may contain one or more of these ingredients: corn starch, croscarmellose sodium, sodium starch glycolate.

Mixture B	No. of xtals[a]	Area.xtals (μm^2)[b]	$Ratio_{Obs}$ (%)[c]
D-Glucose	32	8.10	3.0
D-Sucrose	31	10.71	3.9
D-Maltose monohydrate	92	25.13	9.2
L-Arabinose	33	10.20	3.8
L-Ascorbic acid	74	21.68	8.0
D-Galactose	52	12.70	4.7
D-Trehalose dihydrate	255	73.19	26.9
D-Xylose	145	37.34	13.7
L-Rhamnose monohydrate	286	72.80	26.8
Mixture C	No. of xtals[a]	Area.xtals (μm^2)[b]	$Ratio_{Obs}$ (%)[c]
L-Glutamic acid	172	45.66	15.7
L-Alanine	53	15.08	5.2
L-Tyrosine	215	48.85	16.8
L-Serine	51	12.66	4.4
L-Valine	197	50.41	17.4
L-Cysteine	207	55.12	19.0
L-Threonine	68	15.78	5.4
L-Aspartic acid	57	16.04	5.5
L-Glutamine	101	45.66	10.5

Table S6 Compositional analysis in mixture B and C. [a]'No. of xtals' represents the number of crystals identified for each component. [b]the crystal areas were converted from $pixel^2$ to μm^2 by the pixel sizes and magnification. [c]$Ratio_{Obs}$ was calculated by dividing the ratio of the total area of one component to the total area of all crystals.

Aspirin tablet	No. of xtals[a]	Area.xtals (μm^2)[b]	$Ratio_{Obs}$ (%)[c]
Aspirin[d]	472	418.56	36.6
Dextrose excipient[e]	103	94.82	8.3
Saccharin sodium	4	5.23	0.5
Non-xtals	715	626.00	54.7
Acetaminophen tablet	No. of xtals[a]	Area.xtals (μm^2)[b]	$Ratio_{Obs}$ (%)[c]
Acetaminophen	652	667.72	90.2
Others	56	72.82	9.8

Table S7 Compositional analysis in Aspirin and Acetaminophen tablet. [a]'No. of xtals' represents the number of crystals identified for each component. [b]the crystal areas were converted from $pixel^2$ to μm^2 by the pixel sizes and magnification. [c]$Ratio_{Obs}$ was calculated by dividing the ratio of the total area of one component to the total area of all crystals.[d]contains two polymorphs of aspirin. [e]contains crystals of α-D-glucose, β-D-glucose and α-D-glucose monohydrate.

Mixture C	No. merged dataset	Res. (Å)	Completeness (%)[a]	No. of unique reflections	No. of observed reflections	R_1 (%)[b]
L-Glutamic acid	2	0.75	89.4	838	3120	18.2
L-Alanine	2	0.75	93.3	596	2050	11.5
L-Tyrosine	3	0.75	89.8	1093	6240	12.6
L-Serine	2	0.75	82.2	559	2245	15.5
L-Valine	2	0.75	49.4	803	2936	15.2
L-Cysteine	2	0.75	92.4	731	2676	17.2
L-Threonine	2	0.75	83.0	676	2709	13.2
L-Aspartic acid	4	0.75	91.4	647	2663	21.5

L-Glutamine	3	0.75	98.2	915	4447	13.5
Aspirin tablet	No. merged dataset	Res. (Å)	Completeness (%)[a]	No. of unique reflections	No. of observed reflections	R_1 (%)[b]
Asprin (form 1)	4	0.72	83.3	1855	14444	19.6
Asprin (form 2)	3	0.92	85.2	958	5314	21.7
β-D-Glucose	2	0.70	94.5	1280	7973	19.9
α-D-Glucose monohydrate	4	0.71	81.6	1147	8443	15.2
Acetaminophen tablet	No. merged dataset	Res. (Å)	Completeness (%)[a]	No. of unique reflections	No. of observed reflections	R_1 (%)[b]
Acetaminophen	3	0.70	96.0	2088	10643	19.6

Table S8 MicroED refinement statistics for the crystals structures solved from the automatic SerialEM data collection in Mixture C-the mixture containing amino acids, Aspirin tablet, and Acetaminophen tablet. Structures were solved *ab initio* by SHELXT and refined by SHELXL following automatic processing by the implemented Python script used in this study. [a]Completeness of the merged datasets by XSCALE. [b]R_1 from SHELXL refinement.

Mixture A	CSD/ICSD entry	Space group	a	b	c	α	β	γ
Sodium bicarbonate	26933	$P\ 1\ 2_1/n\ 1$ (14)	7.51	9.70	3.53	90.0	93.0	90.0
Sodium sulfate	136895	F d d d (70)	5.86	12.31	9.82	90.0	90.0	90.0
Potassium sulfate	79777	P m c n (62)	5.75	10.04	7.45	90.0	90.0	90.0
Magnesium sulfate heptahydrate	16111	$P\ 2_1\ 2_1\ 2_1$ (19)	11.87	12.00	6.86	90.0	90.0	90.0
Sodium chloride	29929	F m -3 m (225)	5.63	5.63	5.63	90.0	90.0	90.0
Calcium gluconate	YUXDED	C 2 (5)	15.87	5.88	9.16	90.0	99.6	90.0
Sodium citrate dihydrate	UMOGAE	C 2/c (15)	15.70	12.50	11.28	90.0	103.6	90.0
Calcium acetate monohydrate	CEJLIM	P-1 (2)	6.75	11.08	11.78	116.0	92.0	97.0
Magnesium acetate tetrahydrate	FELGUY01	$P\ 2_1/c$ (14)	4.81	11.99	8.55	90.0	95.4	90.0
Mixture B	CSD entry	Space group	a	b	c	α	β	γ
D-Glucose	GLUCSA	$P\ 2_1\ 2_1\ 2_1$ (19)	10.36	14.84	4.97	90.0	90.0	90.0
D-Sucrose	SUCROS03	$P\ 2_1$ (4)	10.86	8.70	7.76	90.0	103.0	90.0
D-Maltose monohydrate	MALTOS11	$P\ 2_1$ (4)	4.87	15.08	10.70	90.0	97.1	90.0
L-Arabinose	ABINOS01	$P\ 2_1\ 2_1\ 2_1$ (19)	6.51	19.45	4.84	90.0	90.0	90.0
L-Ascorbic acid	LASCAC12	$P\ 2_1$ (4)	6.39	6.26	17.13	90.0	99.4	90.0
D-Galactose	ADGALA01	$P\ 2_1\ 2_1\ 2_1$ (19)	5.94	7.87	15.80	90.0	90.0	90.0
D-Trehalose dihydrate	TREHAL01	$P\ 2_1\ 2_1\ 2_1$ (19)	12.23	17.89	7.60	90.0	90.0	90.0
D-Xylose	ZZZSEA	$P\ 2_1\ 2_1\ 2_1$ (19)	9.10	12.10	5.60	90.0	90.0	90.0
L-Rhamnose monohydrate	RHAMAH01	$P\ 2_1$ (4)	7.91	7.92	6.67	90.0	95.6	90.0
Mixture C	CSD entry	Space group	a	b	c	α	β	γ
L-Glutamic acid	LGLUAC	$P\ 2_1\ 2_1\ 2_1$ (19)	5.17	17.34	6.95	90.0	90.0	90.0
L-Alanine	LALNIN	$P\ 2_1\ 2_1\ 2_1$ (19)	6.03	12.34	5.78	90.0	90.0	90.0
L-Tyrosine	LTYROS10	$P\ 2_1\ 2_1\ 2_1$ (19)	6.91	21.12	5.83	90.0	90.0	90.0
L-Serine	LSERIN01	$P\ 2_1\ 2_1\ 2_1$ (19)	8.60	9.35	5.62	90.0	90.0	90.0
L-Valine	LVALIN	$P\ 2_1$ (4)	9.71	5.27	12.06	90.0	90.8	90.0
L-Cysteine	LCYSTN34	$P\ 2_1\ 2_1\ 2_1$ (19)	5.42	8.11	12.15	90.0	90.0	90.0
L-Threonine	LTHREO	$P\ 2_1\ 2_1\ 2_1$ (19)	13.61	7.74	5.14	90.0	90.0	90.0
L-Aspartic acid	LASPRT	$P\ 2_1$ (4)	7.62	6.98	5.14	90.0	99.8	90.0
L-Glutamine	GLUTAM	$P\ 2_1\ 2_1\ 2_1$ (19)	16.01	7.76	5.10	90.0	90.0	90.0

Table S9 Reference unit cell parameters for the compounds in Mixture A-C.

Aspirin tablet	CSD entry	Space group	a	b	c	α	β	γ
Asprin (form 1)	ACSALA01	P 2_1/c (14)	11.43	6.59	11.4	90.0	95.7	90.0
Asprin (form 2)	ACSALA15	P 2_1/c (14)	12.15	6.51	11.37	90.0	111.6	90.0
α-D-Glucose	GLUCSA	P 2_1 2_1 2_1 (19)	10.36	14.84	4.97	90.0	90.0	90.0
β-D-Glucose	GLUCSE	P 2_1 2_1 2_1 (19)	9.29	12.65	6.70	90.0	90.0	90.0
α-D-Glucose monohydrate	GLUCMH11	P 2_1 (4)	8.80	5.09	9.71	90.0	97.7	90.0
Saccharin sodium	MGSACD11	P-1 (2)	7.27	11.60	15.13	72.5	87.7	84.0
**Acetaminophen tablet**	**CSD entry**	**Space group**	**a**	**b**	**c**	**α**	**β**	**γ**
Acetaminophen	HXACAN03	P 2_1/n	11.73	9.38	7.11	90.0	97.5	90.0

Table S10 Reference unit cell parameters for the compounds in Aspirin and Acetaminophen tablet.

3. Figures

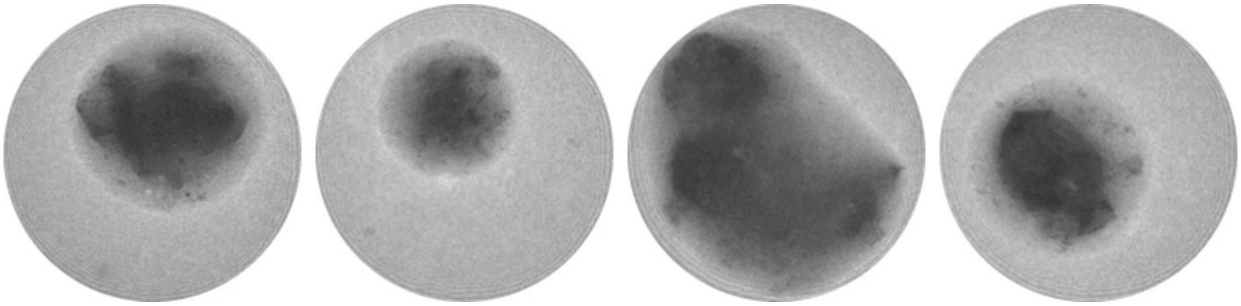

Figure S1 Example of crystals from Mixture A which appears to have “melted”, from which there was a lower degree of successfully indexed data set. There are visually two areas of different contrast, a darker particle shaped inner area surrounded by a lighter drop-like outer area. It seems likely that the crystals partially decomposed before being frozen in the microscope resulting in poor diffraction.

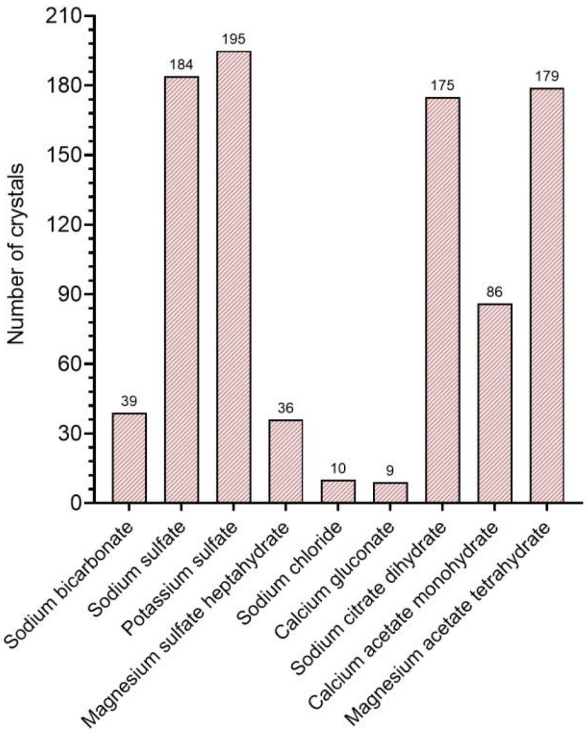

Figure S2 Compound identification in Mixture A. The bars represent the number of crystals identified for each composition in mixture A. Despite the smaller number of data sets used, 913 in total, all compounds could be identified including the compounds with the lowest relative mass of 3% of the total weight of the composition.

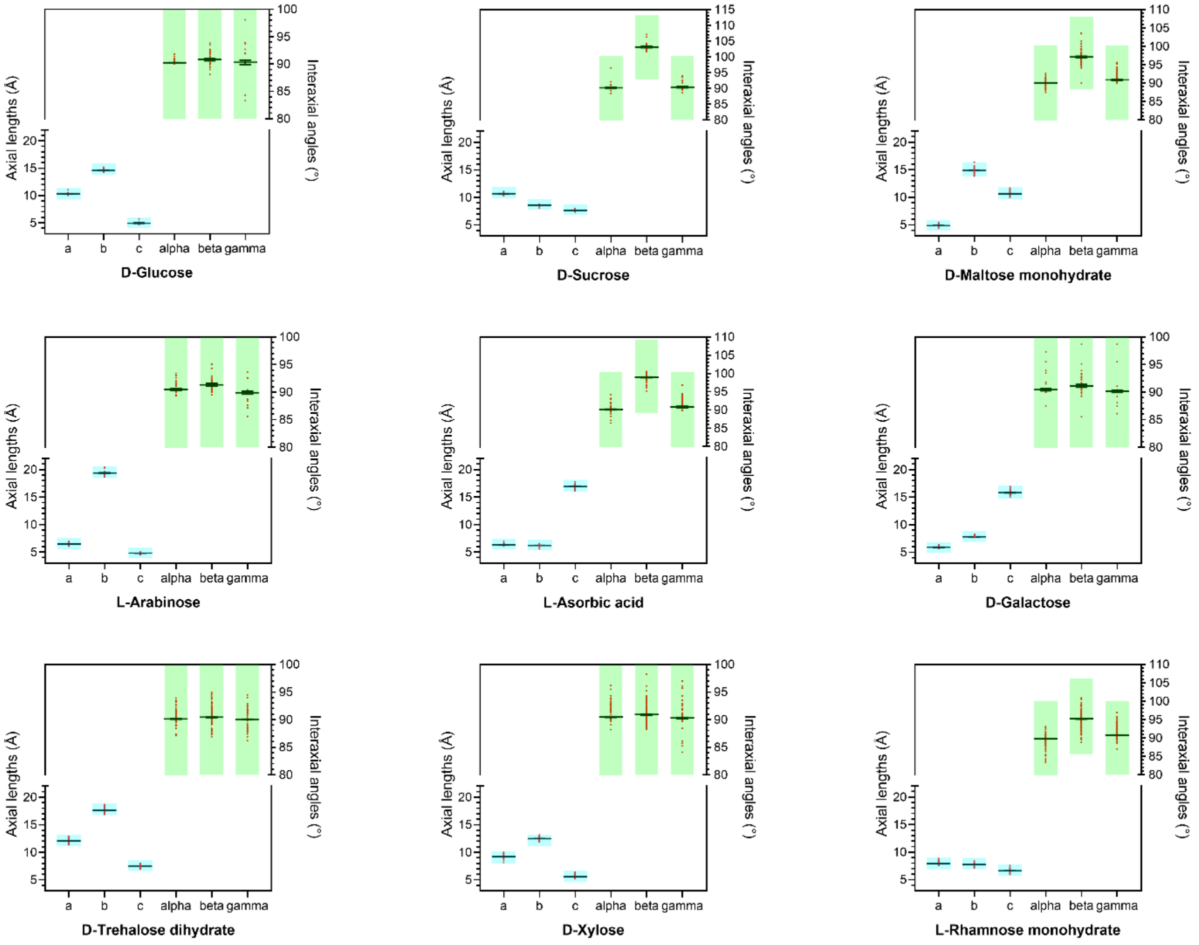

Figure S3 Unit cell parameters from processing in mixture B showing unit cell parameters plotted as red dots, mean and standard deviations plotted as black lines, and the tolerance bars are shown by blue and green boxes. The experimental unit cell parameters fall well within the selected tolerance limits which ensure an accurate unit cell determination. There is no overlap of the six unit cell parameters between the space groups and the structure assignments were conclusive.

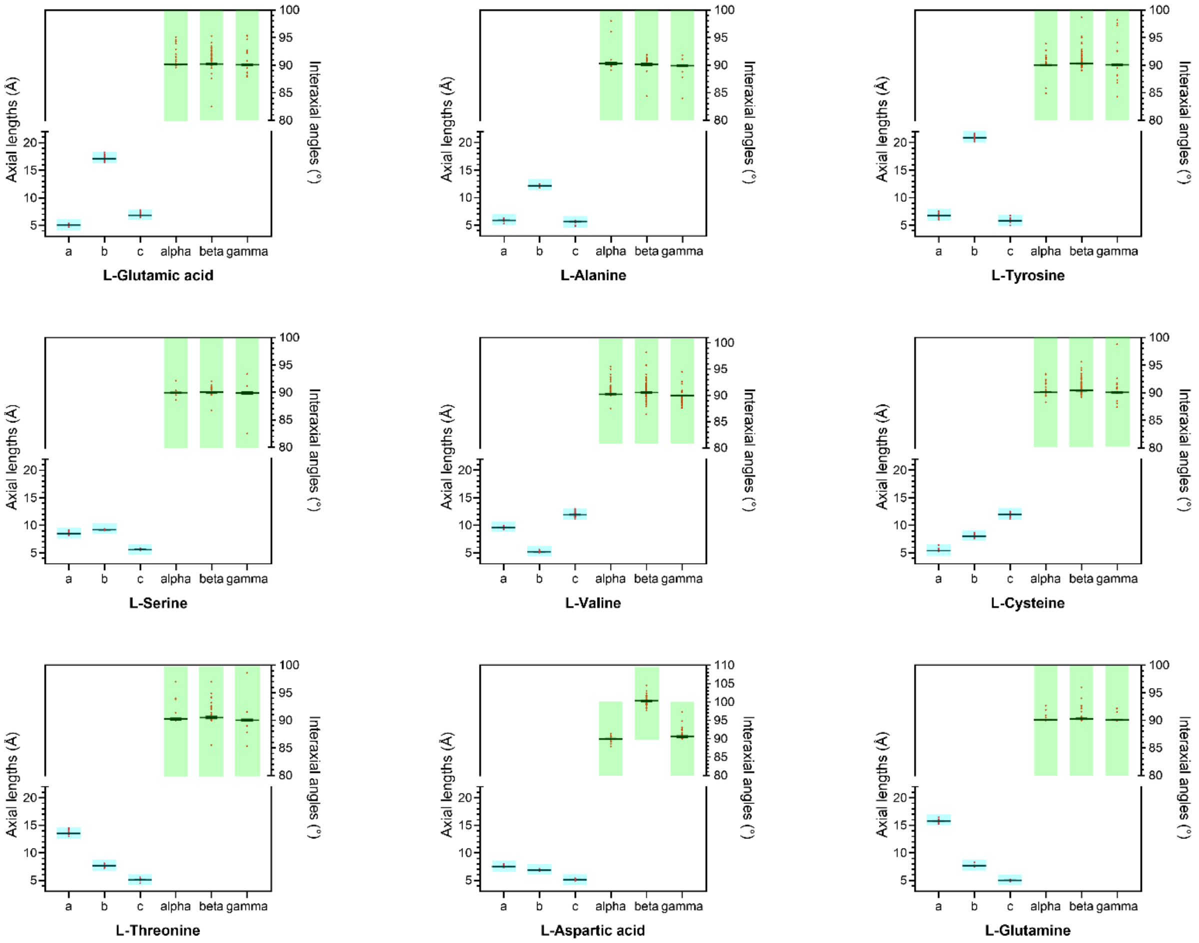

Figure S4 Unit cell parameters from processing in mixture C showing unit cell parameters plotted as red dots, mean and standard deviations plotted as black lines, and the tolerance bars are shown by blue and green boxes. The experimental unit cell parameters fall well within the selected tolerance limits which ensure an accurate unit cell determination. There is no overlap of the six unit cell parameters between the space groups and the structure assignments were all conclusive.

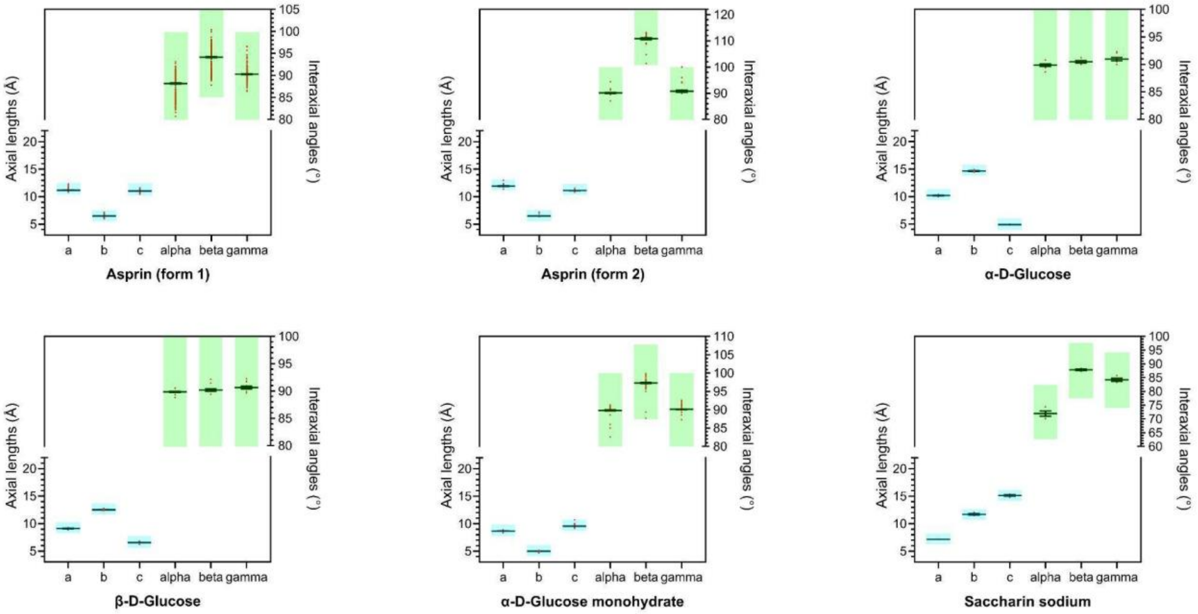

Figure S5 Unit cell parameters from processing in aspirin tablet showing unit cell parameters plotted as red dots, mean and standard deviations plotted as black lines, and the tolerance bars are shown by blue and green boxes. The experimental unit cell parameters fall well within the selected tolerance limits which ensure an accurate unit cell determination. There is no overlap of the six unit cell parameters between the space groups and the structure assignments were all conclusive.

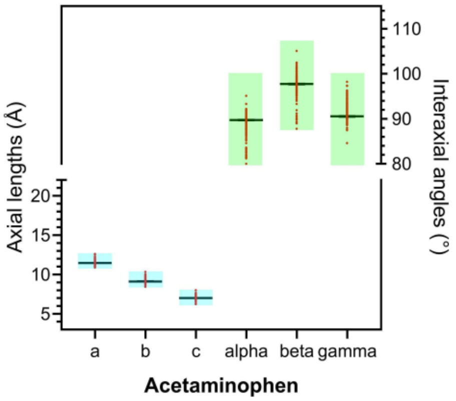

Figure S6 Unit cell parameters from processing in acetaminophen tablet showing unit cell parameters plotted as red dots, mean and standard deviations plotted as black lines, and the tolerance bars are shown by blue and green boxes. The experimental unit cell parameters fall well within the selected tolerance limits which ensure an accurate unit cell determination. There is no overlap of the six unit cell parameters between the space groups and the structure assignments were all conclusive.

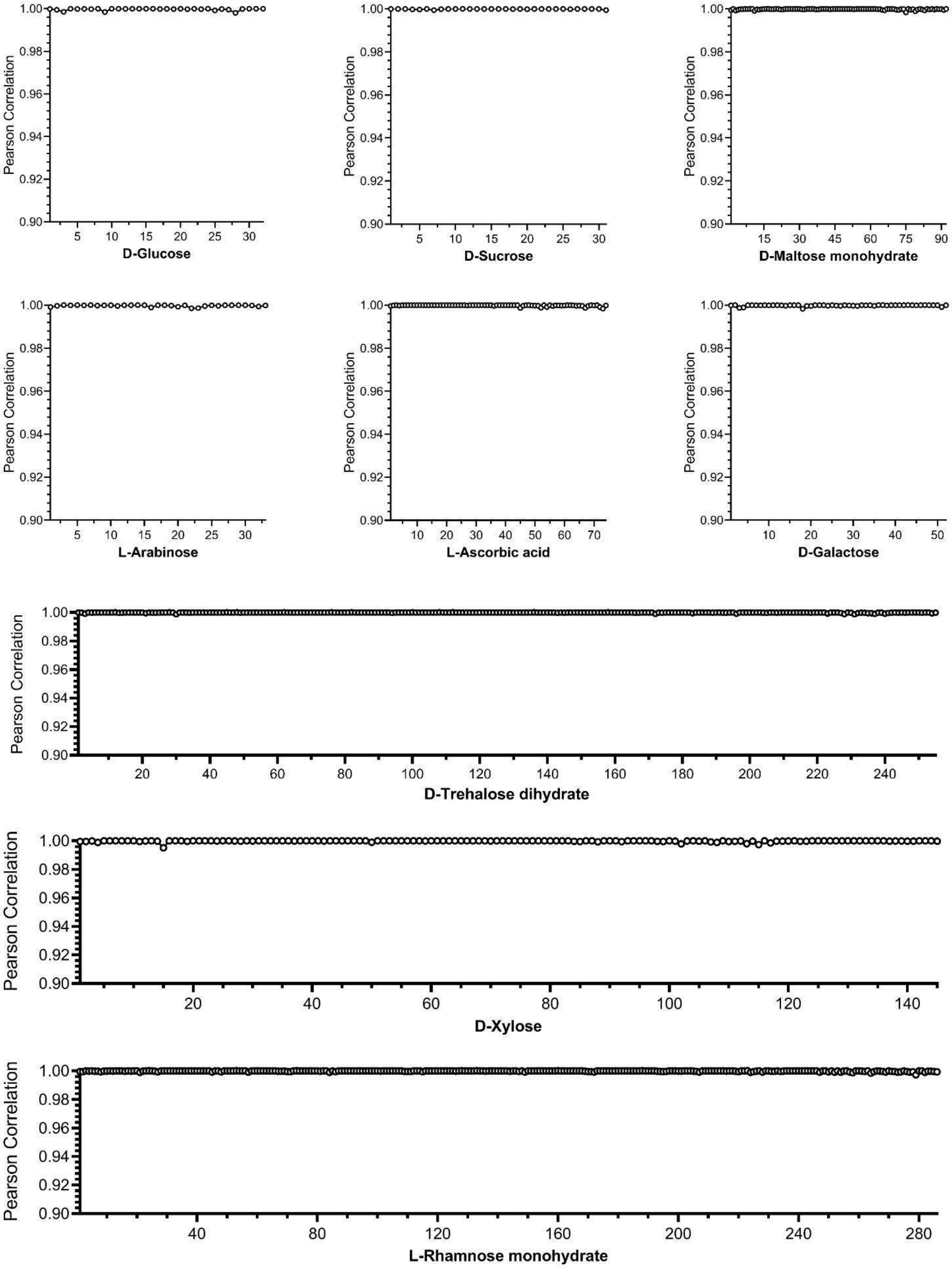

Figure S7 Pearson correlation plot of unit cell parameters from processing in mixture B and the reference unit cell parameters.

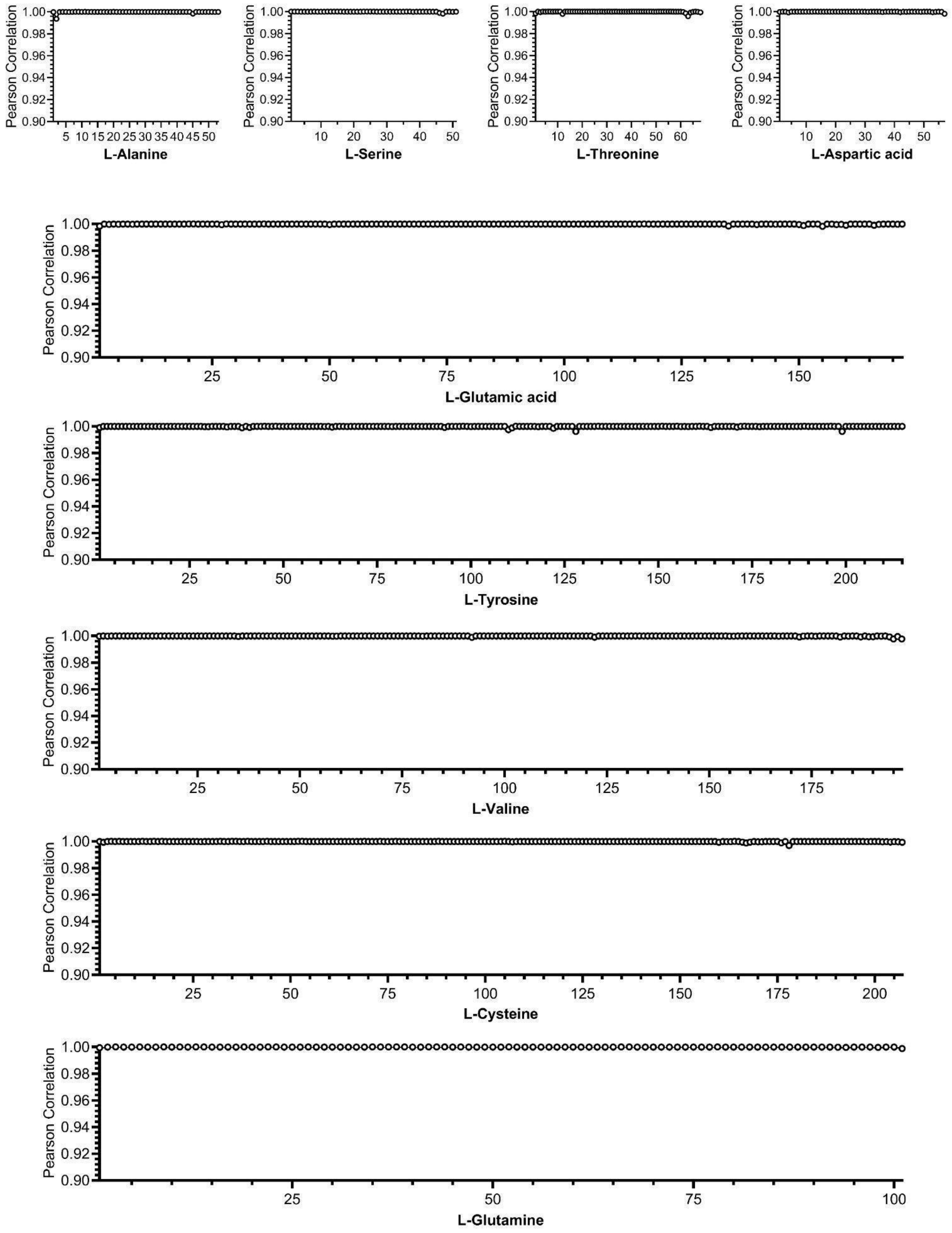

Figure S8 Pearson correlation plot of unit cell parameters from processing in mixture C and the reference unit cell parameters.

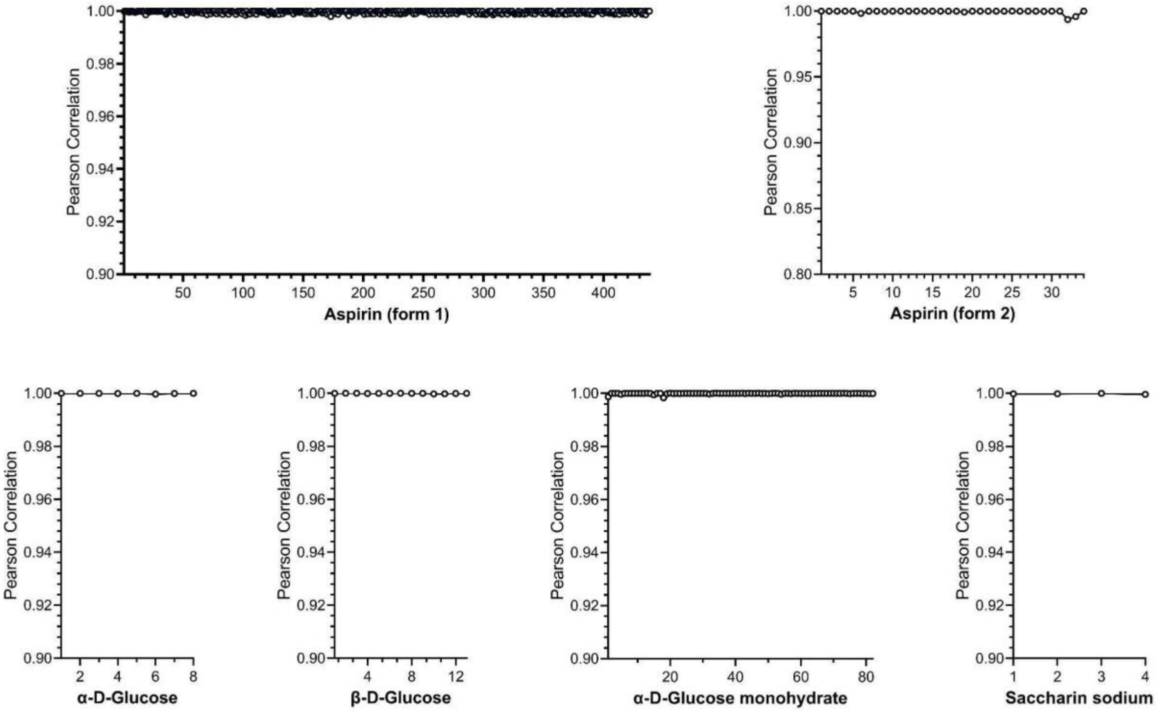

Figure S9 Pearson correlation plot of unit cell parameters from processing in aspirin tablet and the reference unit cell parameters.

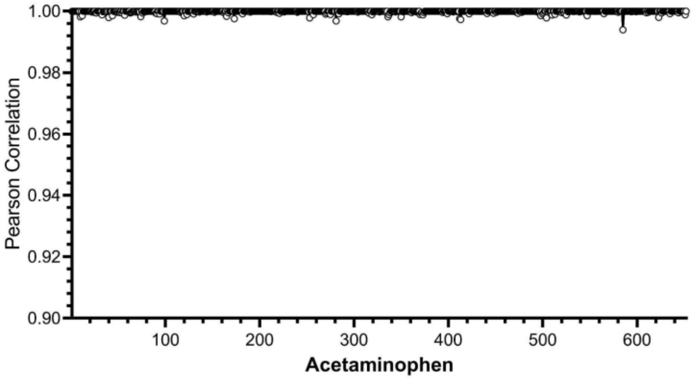

Figure S10 Pearson correlation plot of unit cell parameters from processing in acetaminophen tablet and the reference unit cell parameters.
